# Global trends in surgical approach to neonatal brachial plexus palsy: a systematic review

**DOI:** 10.3389/fsurg.2024.1359719

**Published:** 2025-01-06

**Authors:** Michael F. Levidy, Amanda Azer, Jasmine Shafei, Nivetha Srinivasan, Jasmine Mahajan, Shivani Gupta, George Abdelmalek, Krittika Pant, Kunj Jain, Yash Shah, Zheshi Zheng, Johnny Chuieng-Yi Lu, Tommy Nai-Jen Chang, Alice Chu, Aleksandra McGrath

**Affiliations:** ^1^ Department of Orthopedic Surgery, Rutgers New Jersey Medical School, Newark, NJ, United States; ^2^ Department of Plastic and Reconstructive Surgery, Linkou Medical Center and Chang Gung Medical College and Chang Gung University, Chang Gung Memorial Hospital, Taoyuan, Taiwan; ^3^ Department of Clinical Sciences, Umeå University, Umeå, Sweden; ^4^ Department of Surgical and Perioperative Sciences, Faculty of Medicine, Umeå University, Umeå, Sweden

**Keywords:** brachial plexus, neonatal brachial palsy, trends, nerve transfer, nerve exploration

## Abstract

**Background:**

We analyzed trends in age at surgery and surgical approach over time and geography.

**Methods:**

We performed a systematic review according to PRISMA-IPD guidelines to include individual patient data. Collected data included age at surgery, location of surgery, and surgical approach. The surgical approach was independently categorized as the exploration of the brachial plexus (EBP) or nerve transfer without root exploration (NTwoRE). EBP was defined as exploring the brachial plexus in the supraclavicular fossa and applying a choice of coaptation procedures. NTwoRE included those sourcing donor nerves from, or entirely occurring outside of the plexus without exploring the root of the brachial plexus.

**Results:**

Regression analysis of age at surgery 1985–2020 showed that age at BPBI surgery is rising (*p* < 0.05). Surgery was performed at a younger age in patients from Europe (7.06 ± 7.77 months) and Asia (7.58 ± 5.33 months) than those from North America (10.44 ± 5.01 months) and South America (14.71 ± 4.53 months) (*p* < 0.05). NTwoRE was more common in North America (37%) and least common in Europe (12%). Age at EBP was 7.2 ± 5.77 months, and age at NTwoRE was 15.85 ± 13.18 months (*p* < 0.05). The incidence of NTwoRE is increasing time.

**Conclusions:**

Age at NBPP surgery is increasing over time. Regional differences exist in age at NBPP surgery. Approaches to NBPP surgery that avoid exploration of BP roots are becoming more popular. Age at EBP is lower than age at NTwoRE.

## Introduction

Many factors potentially affect the outcome of neonatal brachial plexus palsy (NBPP), including the timing of diagnosis, severity of the palsy, surgeons’ experiences, timing of the approach, and different surgical approaches. However, the existing literature is often difficult to interpret because of different outcome measures and variability in the severity of plexus injuries. Because NBPP is a rare condition, much of the literature on surgical treatment is of a low level of evidence, with few randomized clinical trials (1). Surgeon preference affects decision-making in the timing and approach to surgery, as do several difficult-to-quantify factors: previous training, exposure to different techniques, volume of practice, and specialty of the surgeon. While some authors favor extensive proximal exploration of the nerve roots of the plexus, others with similar experience prefer nerve transfers without exploring the roots or orthopedic procedures that avoid nerve manipulation entirely (2).

Age at surgery has been investigated for its role in NBPP patient outcomes, with numerous authors providing recommendations on the topic. Gilbert and Tassin recommended surgery before 3 months (3), Chuang et al. before 6 months (4), and Clarke and Curtis before 9 months (5). More recently, a multicenter study from Bauer found no difference in surgical outcomes between groups aged 4.2 months and 10.7 months, while a systematic review from Srinivasan et al. suggested that intervention before 6 months was associated with improved Mallet scores (6, 7).

Initially, the technique of NBPP microsurgery was the exploration of supraclavicular fossa and arduous dissection around the scarred brachial plexus, with selective neurolysis and nerve grafting. Gradually, through the innovation of pioneering surgeons such as Oberlin (8) and Mackinnon (9), close target nerve transfers were developed and became adopted in the treatment of NBPP as well as traumatic adult brachial plexus injuries (10). In recent years, there were reports of NBPP patients with partial palsies, treated entirely with nerve transfers close to the target, without exploring the brachial plexus. The decision-making in the choice of treatment of NBPP is primarily influenced by the severity of the palsy, with total palsy necessitating brachial plexus exploration, preferably performed early, based on the biology of nerve injury in an immature nervous system (11). However, the rationale behind choosing not to explore the plexus in the group of patients with partial palsies has not been studied. Similarly, there are no studies investigating whether the introduction of nerve transfers in NBPP has proceeded uniformly across the world.

The objective of this study is to systematically review individualized patient data (IPD) over the past 40 years of NBPP surgery globally to identify trends in surgical approach and age at surgery over time and by continent. The results offer insight into the impact of influential publications and techniques over time and help practitioners understand influences guiding their international colleagues' thinking.

## Methods

We searched PubMed, Cochrane, Web of Science, and the Cumulative Index to Nursing and Allied Health Literature databases in accordance with PRISMA-IPD guidelines. Boolean searches were performed using the terms “brachial plexus,” “injury,” “palsy,” “nerve plexus,” “upper plexus,” “pediatric,” “surgery,” and others. Search filters were non-full-text, non-human studies, review papers, commentaries, editorials, and non-English papers. Studies were included if they involved surgical treatment of NBPP, included >2 patients, included patient-level data, and included a description of surgery. Studies were excluded if they were irrelevant to NBPP; were case studies or reviews; were not microsurgical; were adult-only; were imaging studies or did not include patient-level data. We contacted authors of otherwise eligible papers missing IPD to request access to the full dataset. Disagreements regarding eligibility were resolved by senior authors AC and AM. The included studies were assessed for risk of bias using the Risk of Bias in Non-Randomized Studies of Interventions (ROBINS)-1 tool (12). Four authors (ML, JS, KJ, and YS) graded the risk of bias. AM and AC resolved conflicts. Designations were made relative to the risk of bias for collected data, not the paper.

Data collected included age at surgery, location of surgery, and surgical approach. The location of surgery was based on continent definitions from the CIA World Factbook (13). The year associated with patient data was taken from the publication date of the article. The surgical approach was independently categorized by both senior authors (AM and AC) as the exploration of the brachial plexus (EBP) or nerve transfer without root exploration (NTwoRE). EBP was defined as exploring the brachial plexus in the supraclavicular fossa and applying a choice of coaptation procedures such as nerve grafting, neurolysis, or nerve transfers with targets in supraclavicular or infraclavicular fossa such as roots, divisions, and trunks from variety of donors, such as spinal accessory nerve, hypoglossal nerve, and contralateral C7 transfer or pectoral nerves. NTwoRE included those sourcing donor nerves from or entirely occurring outside of the plexus without exploring the root of the brachial plexus, such as intercostal nerve transfer, spinal accessory to suprascapular nerve transfer, Oberlin or MacKinnon procedure, and radial to axillary nerve transfer.

Statistical analysis of age at surgery vs. time was performed using simple linear regression. Age at surgery vs. continent was analyzed using ANOVA and then pairwise comparisons using *t*-tests with adjusted *p*-values, using the methods introduced by Bonferroni, Holm (14), Hochberg (15), Hommel (16), Benjamini and Hochberg (17), and Benjamini and Yekutieli (18). Statistical analysis of the proportion of NTwoRE surgeries over time was performed using logistic regression with a single numerical covariate. The proportion of NTwoRE surgeries in each continent was analyzed using logistic regression with categorical covariates. Statistical significance was defined as *p* < 0.05. Statistical analysis was performed using RStudio version 1.3.1093.

## Results

**Figure 1:**
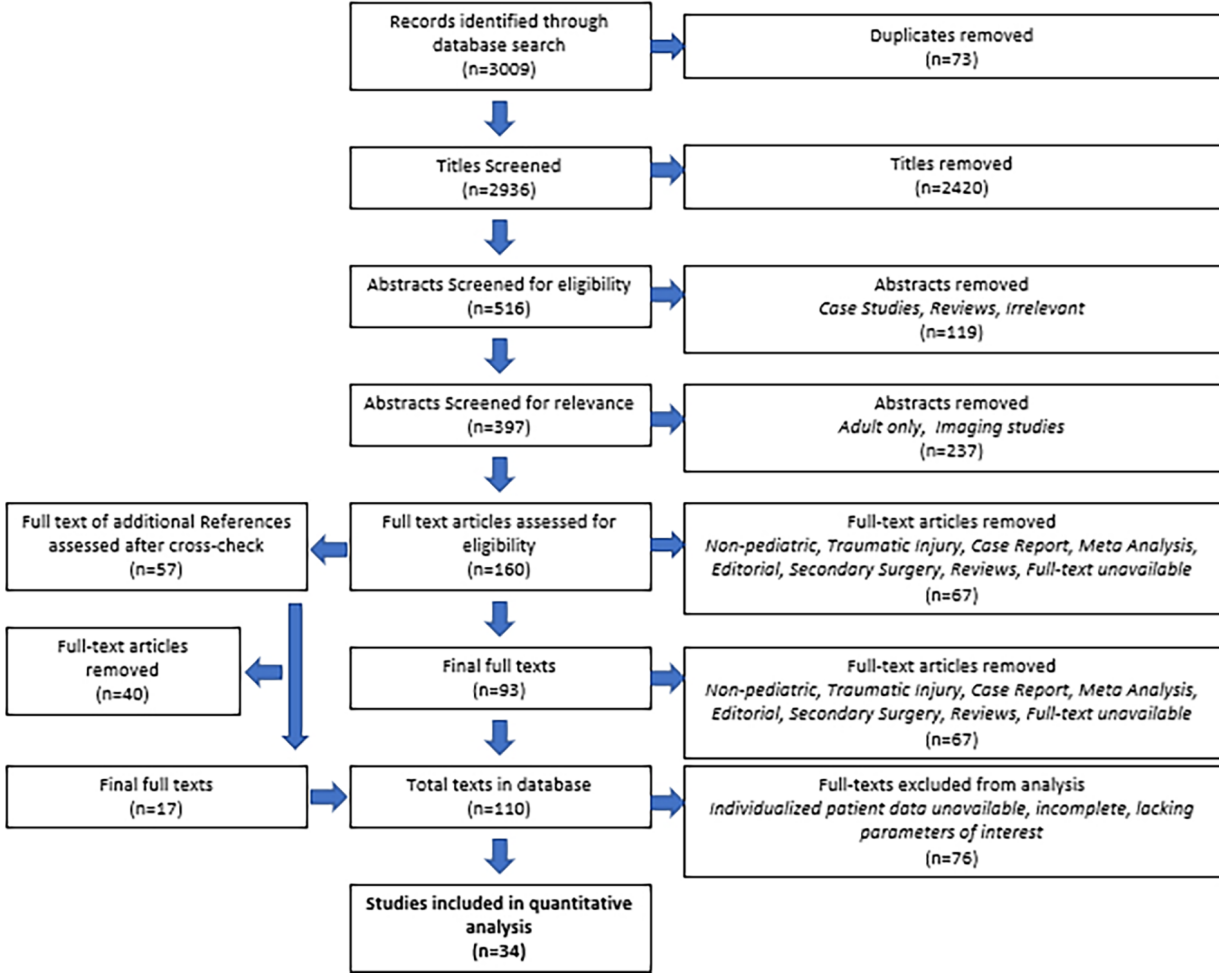
PRISMA workflow.

**Figure 2:**
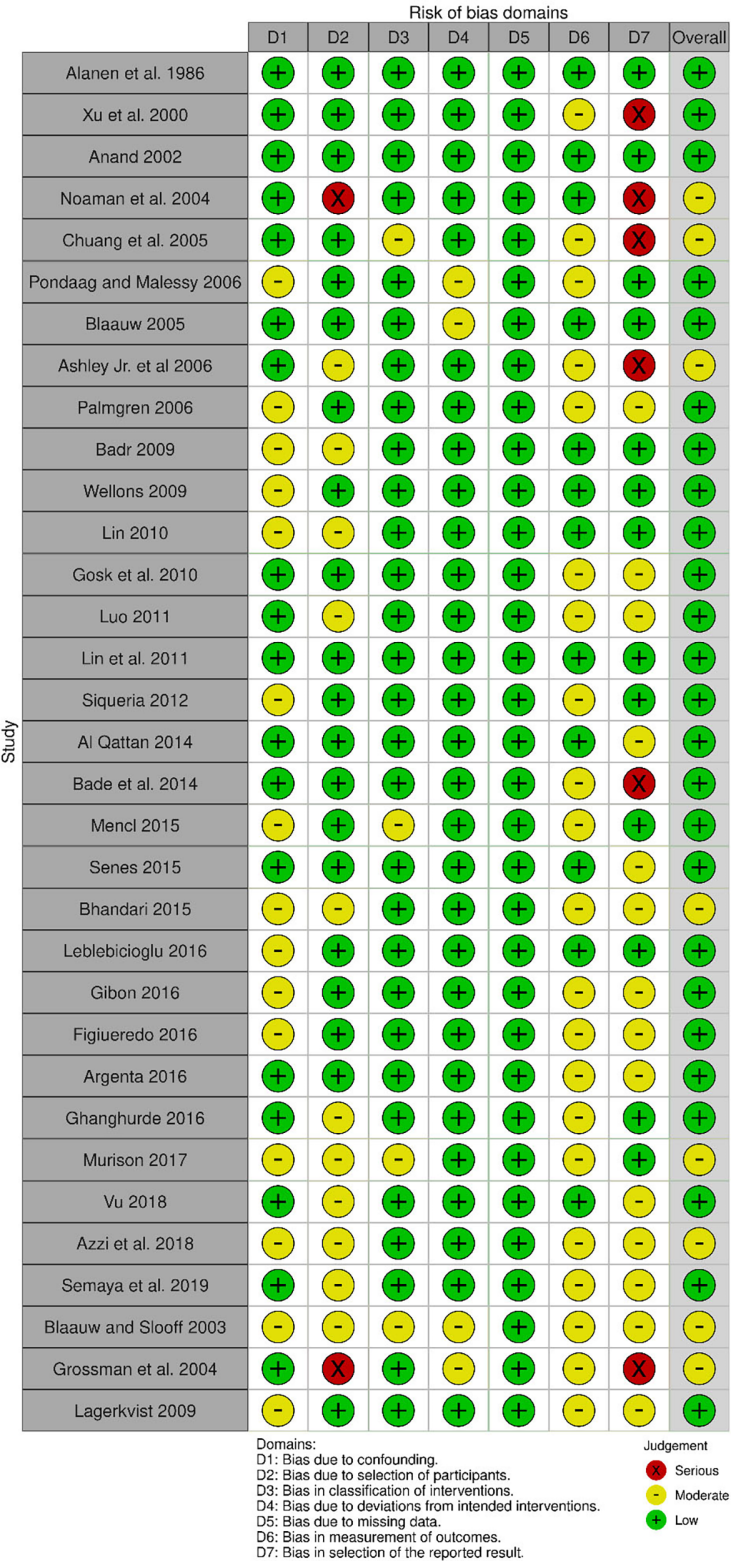
I-Robins assessment of bias.

Starting with 3,009 titles, duplicates were removed until 2,936 titles remained. The screening was performed for the relevance of titles, after which 516 remained. Abstracts of these titles were screened, after which 160 remained. Two authors, ML and JS, then reviewed the full-text manuscripts of these 160 abstracts, and 93 were deemed suitable. Additionally, 57 relevant references were sourced from these 160 full-text manuscripts, cross-checked, and screened. Seventeen additional full-text manuscripts were added this way, for a total of 110 total texts in our database (Figure 1). Of these, 34 contained relevant IPD from a total of 409 patients (Table 1). We found an overall low to moderate risk of bias in all included studies (Figure 2). The bias that existed was largely a result of the selection of outcome measures.

**Table 1:** All included studies in this research study are seen above with the author and publication year.

Study	Total patients	# NTwoRE	# BPE
Alanen et al. (19)	11	0	11
Xu et al. (20)	10	0	10
Anand and Birch (21)	20	0	20
Noaman et al. (22)	7	7	0
Chuang et al. (23)	10	0	10
Pondaag and Malessy (24)	16	0	16
Blaauw et al. (25)	6	0	6
Ashley et al. (26)	5	0	5
Palmgren et al. (27)	14	0	14
Badr et al. (28)	16	0	16
Wellons et al. (29)	20	20	0
Lin et al. (30)	9	0	9
Gosk et al. (31)	8	0	8
Luo et al. (32)	24	0	24
Lin et al. (33)	15	0	15
Siqueria et al. (34)	8	8	0
Al Qattan and El-Sayed (35)	10	10	0
Bade et al. (36)	14	11	3
Mencl et al. (37)	20	0	20
Sénès et al. (38)	5	5	0
Bhandari and Deb (39)	32	0	32
Leblebicioglu et al. (40)	9	0	9
Gibon et al. (41)	9	0	9
Figiueredo et al. (42)	13	13	0
Argenta et al. (43)	5	0	5
Ghanghurde et al. (44)	20	20	0
Murison et al. (45)	5	5	0
Vu et al. (46)	5	0	5
Azzi et al. (47)	8	0	8
Semaya et al. (48)	16	16	0
Blaauw and Slooff (49)	25	0	25
Grossman et al. (50)	11	0	11
Lagerkvist et al. (51)	3	0	3
	409	115	294

The mean patient age at surgery was 9.6 ± 9.2 months. The number of patients and ages at surgery in each continent are shown in Table 2. The surgical approach was NTwoRE in 115 cases and EBP in 294 cases. The number of patients and surgical approach in each continent are shown in Table 2. The average age of an EBP exploration was 7.2 ± 5.77 months, which was significantly different from the average age of an NTwoRE exploration of 15.85 ± 13.18 months (*p* < 0.05).

**Table 2:** Summary statistics for age at surgery in months vs. continent.

	Number of patients	Avg age (STDEV)
Africa‡	16	40.56 (13.94)
Asia*	139	7.58 (5.33)
Europe*	149	7.06 (7.77)
South America†	21	14.71 (4.53)
North America†	84	10.44 (5.01)
Total	409	9.63

NTwoRE, nerve transfer without root exploration; EBP, brachial plexus exploration at the root level.

Ages at surgery in continents marked with different symbols *, †, and ‡ are significantly different *p* < 0.05.

Ages at surgery in continents marked with the same symbols *, †, and ‡ are not significantly different.

**Figure 3:**
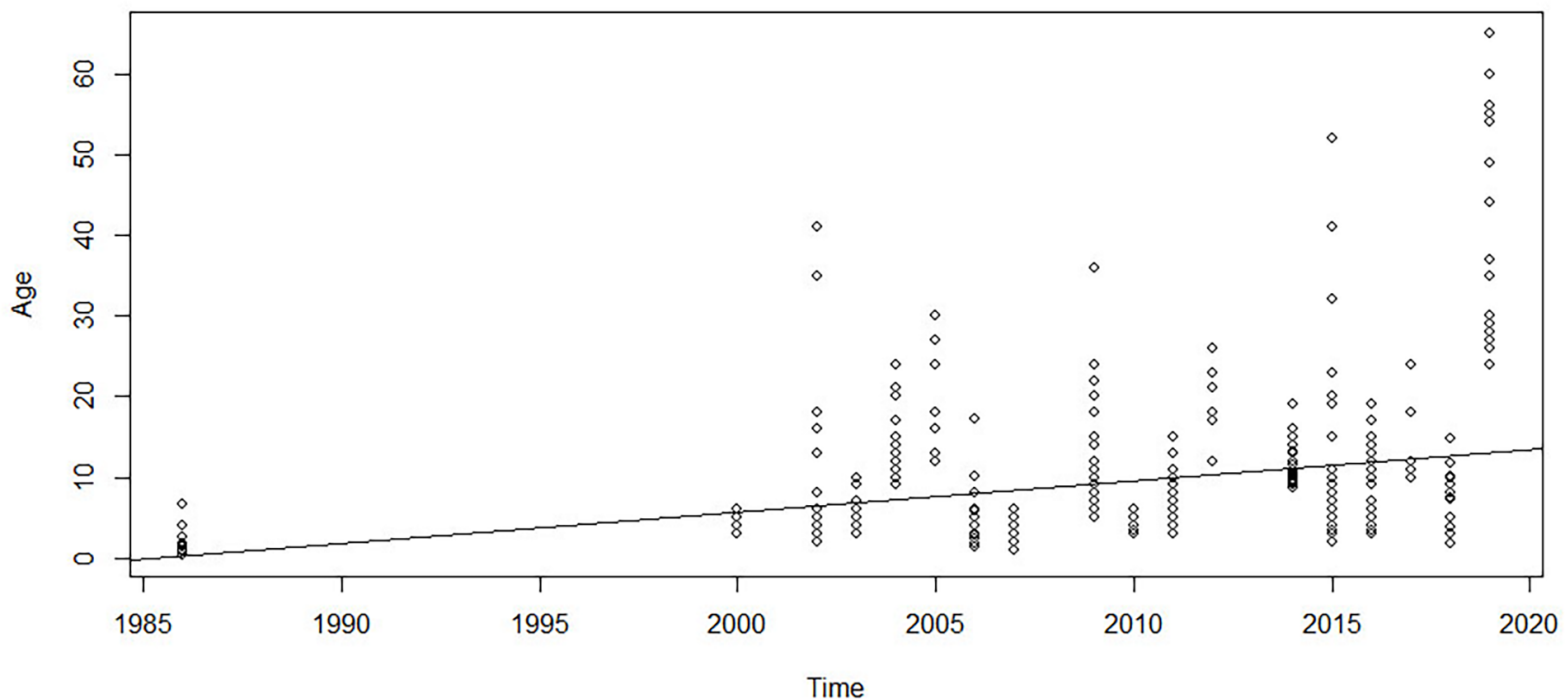
*Y*-axis patient age at surgery in months vs. *x*-axis year.

**Figure 4:**
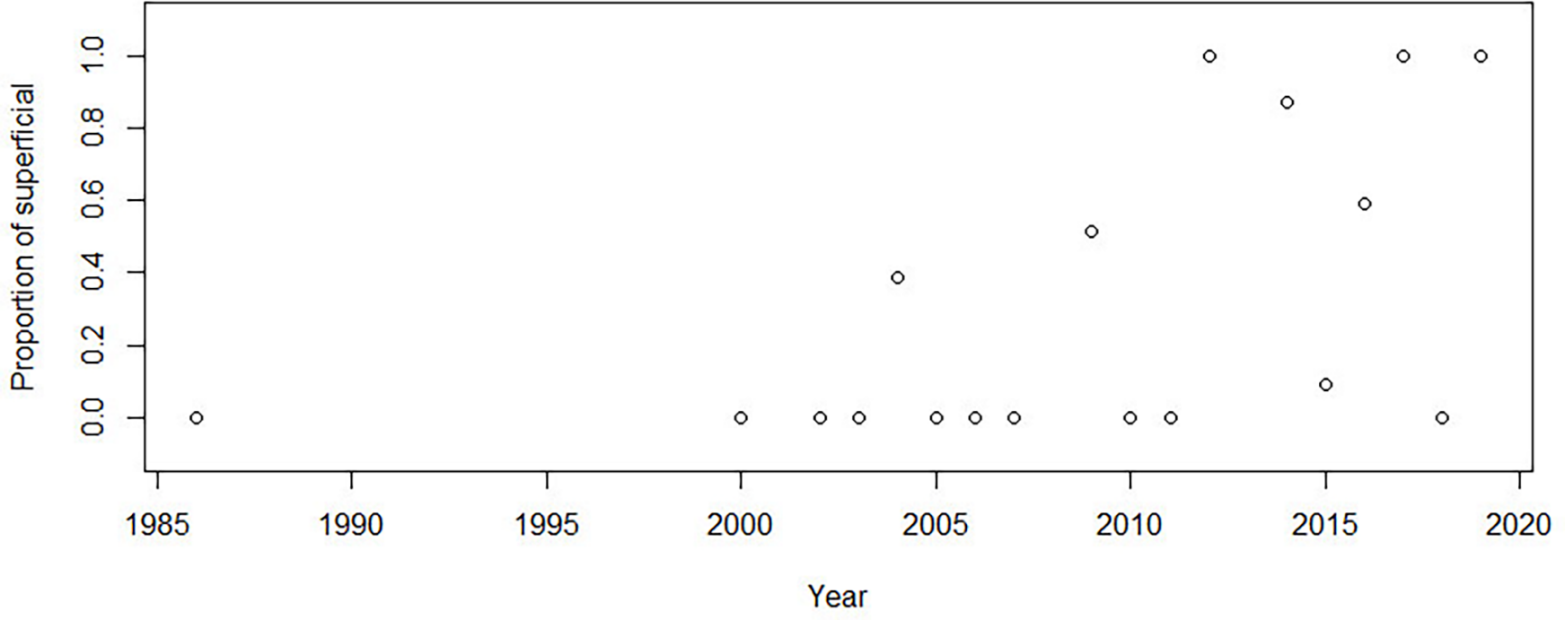
*Y*-axis proportion of surgeries that were nerve transfers without root exploration (ntwoRE) vs. *x*-axis year.

Simple linear regression was used to test if the years from 1984 to 2020 significantly predicted the age at surgery of patients undergoing brachial plexus surgery. The fitted regression model was as follows: age at surgery = −772.14 + 0.389 × (year of surgery). The overall regression was statistically significant [*R*^2^ = 0.075, F (1, 407), *p* < 0.05, Figure 3]. It was found that the year of surgery significantly predicted age at surgery (*β* = 0.382, *p* < 0.01), suggesting that there is a trend of increasing age at surgery over the years. The relationship between year and proportion of cases that were NTwoRE was analyzed using logistic regression. The odds of NTwoRE occurring increased with each year starting in 1985 (*p* < 0.05, Figure 4).

**Figure 5:**
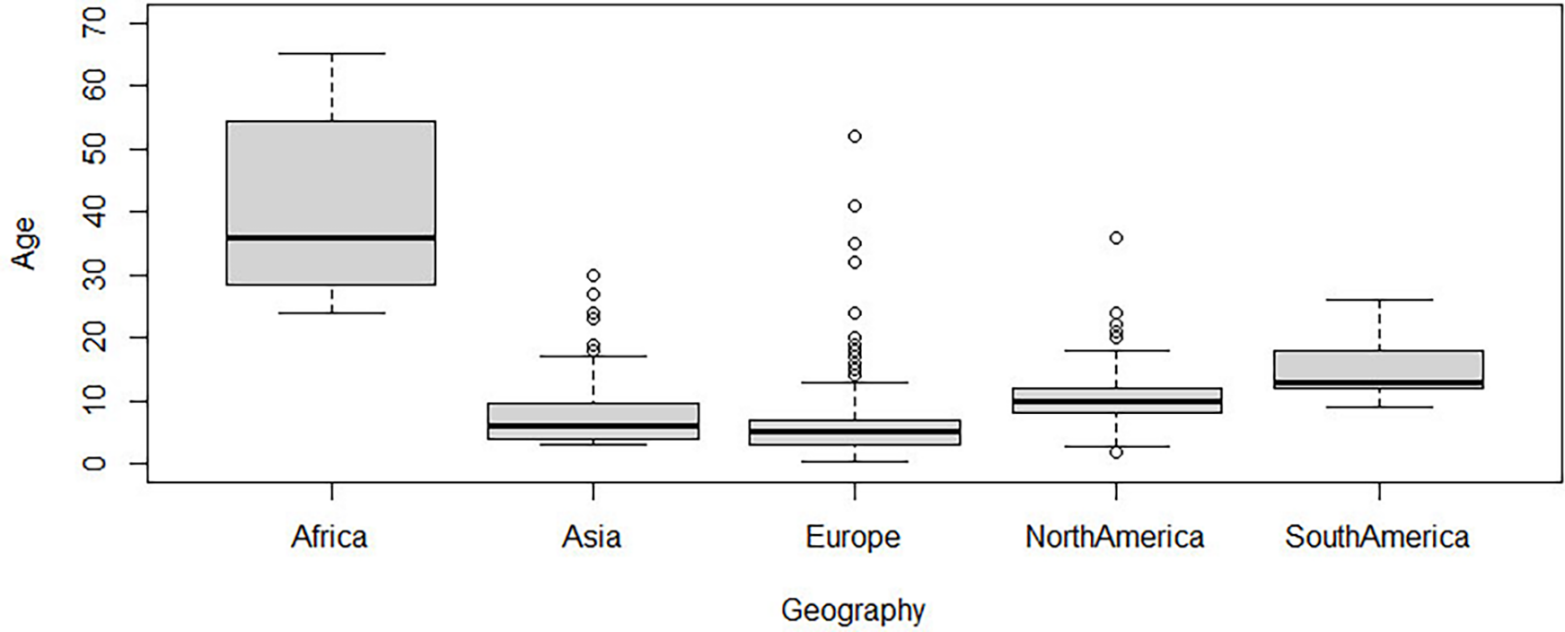
*Y*-axis patient age at surgery in months vs. *x*-axis continent.

**Figure 6:**
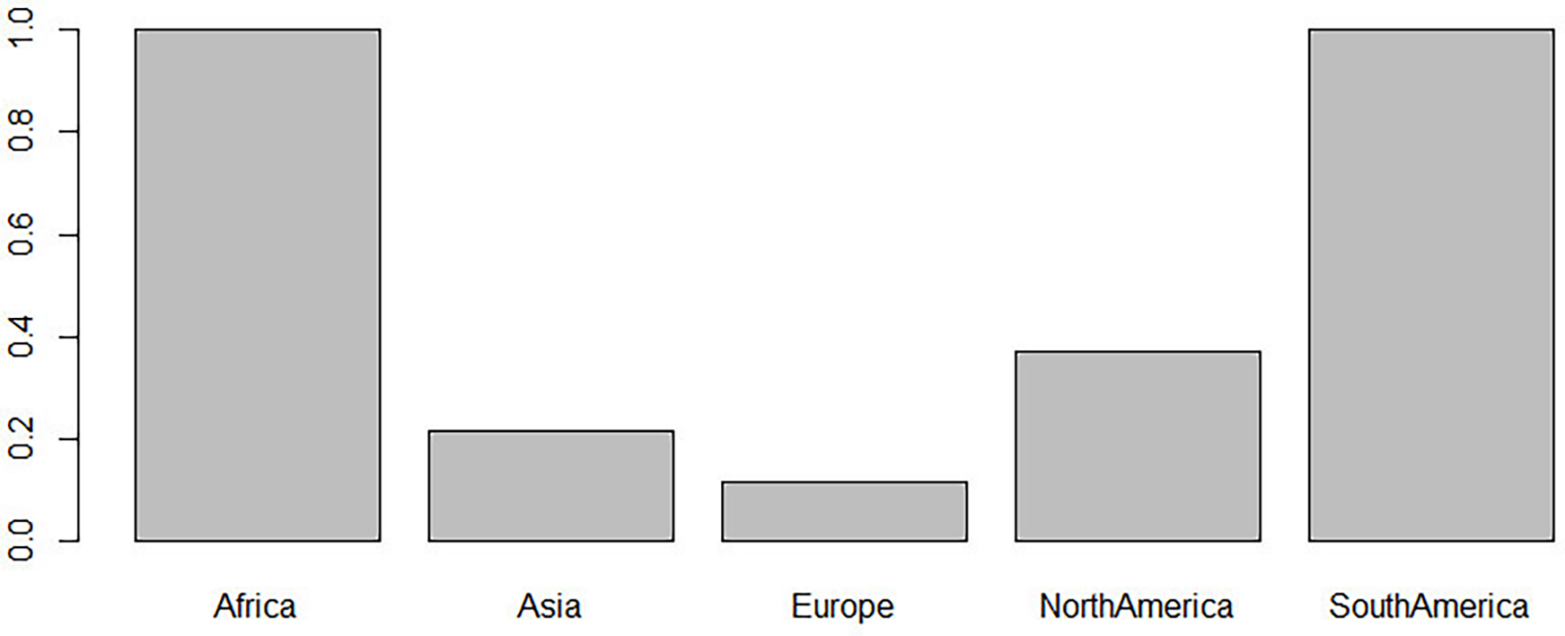
*Y*-axis proportion of nerve transfers without root exploration (ntwoRE) surgery vs. *x*-axis continent.

Comparisons between ages at surgery in different continents were tested by ANOVA with *ad hoc* pairwise *t*-tests using pooled standard deviation. The average age at surgery was youngest in Europe (7.06 ± 7.77 months) and Asia (7.6 ± 5.33 months) and significantly older in North America (10.44 ± 5.01 months) and South America (14.71 ± 4.53 months). Age at surgery in Africa (40.56 ± 13.94) was still higher than that in North and South America. The pairwise comparison proved that those differences were significant (*p* < 0.05, Figure 5, Table 3). Surgical approaches across continents were compared using logistic regression. The odds of an NTwoRE approach occurring in North America (37%) were higher than occurring in Europe (12%) (*p* < 0.05, Figure 6, Table 3).

**Table 3:** Summary statistics for surgical approach vs. continent.

	EBP	NTwoRE	Total
Africa§	0	16	16
Asia*	109	30	139
Europe†	132	17	149
North America‡	53	31	84
South America§	0	21	21
Total	294	115	409

NtwoRE, nerve transfer without root exploration; EBP, exploration of the brachial plexus.

Surgical approaches in continents marked with different symbols * and † are significantly different from one another *p* < 0.05.

Surgical approaches in continents marked with § are not significantly different from any other continent.

## Discussion

In 1900, Thorburn described the first successful surgical repair of brachial plexus injury; however, surgical treatment failed to gain popularity (52, 53). It was not until 1967 that Millesi would pioneer the use of microsurgery in the treatment of brachial plexus injury with nerve grafting (54). In the 1980s, others such as Gilbert (55, 56) and Kawabata (57) applied microsurgical techniques to NBPP repair, with impressive results. Since then, the field has continued to evolve. This investigation was undertaken to describe how and where this evolution occurred. We documented a significant increase in age at surgery over time, changes in preferred surgical targets over time, and marked geographical differences in the management of BPBI.

Age at surgery has long been a focus among BPBI surgeons. Some favor an early (<3 months) intervention, citing potential advantages related to the biology of nerve regeneration, others seek to delay surgery, while others forego primary nerve surgery entirely (3–7, 58–62). Still, others suggest that a 3-month cutoff helps secure parental consent for potentially curative nerve surgery before limited non-operative recovery causes hesitance, introducing ethical dilemmas in the light of equivocal and low-quality evidence (1, 2, 44, 63, 64). In this systematic review, we found that, in data published over the past several decades, age at surgery has increased and the technique is more often NTwoRE. We also found that almost half of all patients from studies containing individual patient data (IPD) were treated with nerve transfers performed close to the target without exploring the supraclavicular fossa and that the proportion of patients treated this way is steadily increasing. Based on published data, the trends are moving from nerve grafts through (63) combined methods to abandoning exploration at the root level. Further, our data show variation in NBPP surgical approaches across the globe. Surgeons in North America tend to intervene at older ages and use NTwoRE nerve transfers more than surgeons in Europe and Asia.

The driving forces behind these geographical and temporal trends are not immediately apparent. In some of the included studies, authors included their rationale for abandoning supraclavicular exploration. It is important to note that in all cases, the patients had partial palsies, where potentially viable nerve stumps available for grafting could have been present and utilized if patients presented early enough. The reasons for choosing the nerve transfer approach were late presentation and recovery of shoulder function with concomitant absence of biceps function, initially non-consenting parents changing their minds, unsuccessful primary nerve surgery, and parents' choice between supraclavicular exploration or multiple nerve transfers. Others chose not to provide a reason, revealing a fundamental challenge with all NBPP studies, the large amount of surgeon's discretion. One example is the choice of outcome measures. Numerous grading systems are used: MRC force ratings, Raimondi scale, mallet, active movement scale (AMS), Gilbert shoulder score, assisting hand assessment, and more. Recently, the iPLUTO survey attempted to gather consensus on which measure is best (43). Certain outcome measures were identified for inclusion in future NBPP investigations: AROM-d, mallet, and passive ROM. The variability of plexus injury subtypes, follow-up times, and ages at surgery also raises questions as to the comparability of patient groups between studies.

Another factor that may contribute to the trends we documented is the lack of clear radiological parameters to guide diagnosis and approach to NBPP. No systematic reviews examining MRI's utility in diagnosing NBPP yet exist. Previous studies have found that the diagnostic accuracy is modest but varies depending on the location and severity of the injury and thus should remain a supplemental, not diagnostic, or treatment-guiding tool (66–68). Previous systematic reviews for adult plexus injuries concluded that MRI offers modest diagnostic accuracy and may help identify graftable stumps, but surgical exploration should remain the preferred method of diagnosis (69, 70). Given the acceptance of MRI in this treatment-guiding capacity, it is likely that factors surrounding the accuracy, availability, and interpretation of MRI have a significant impact on surgical timing and approach to NBPP. Access to MRI equipment and experienced musculoskeletal radiologists varies throughout the world, and with time (71, 72), which may contribute to the global and temporal trends that we documented. The availability of both factors, along with unambiguous MRI findings suggesting avulsion may convince surgeons to operate sooner.

Geographic trends may stem from the geographically fixed nature of NBPP training programs, whereby proponents of a certain approach train the next generation of surgeons. Another possible cause is geographic variation in the specialty responsible for NBPP surgery—pediatric surgery, plastic and reconstructive surgery, neurosurgery, or orthopedic surgery (43). For example, in the USA and Thailand, NBPP is treated largely by pediatric orthopedic surgeons; however, in France, this is not the case. Centralization of NBPP treatment may also play a role. Countries where NBPP is treated at specialized centers, such as Sweden and the UK, may have different preferences than those where treatment of NBPP is not centralized. Preferences in outcome measures were found to vary geographically by the iPLUTO survey, and this may also drive global preference in surgical approach and age at surgery (43). Differences in health systems between countries could also drive these differences. Later presentation may be more common in developing countries or those with high numbers of uninsured patients, such as the USA, or those with a low density of brachial plexus surgeons. There may be an urgency to make a decision skewed towards NtwoRE in places where follow-up is less likely.

We also found that age at surgery in NTwoRE surgeries was significantly older than the age for proximal plexus explorations. Daly et al. and Tarek et al. found that microsurgical repair via proximal plexus exploration is a viable option in late-presenting NBPP, with a combination of approaches in patients with varying degrees of severity of the palsy (73, 74). However, our findings indicate that surgeons still prefer NTwoRE when managing late-presenting patients (38). Evidence supports the notion that NTwoRE such as the Oberlin I procedure is useful in the late presentation of *traumatic* brachial plexus injury (75). Whether this finding translates to NBPP is yet unclear, but surgeons may be applying it thusly—our findings show that surgeons are performing more close target procedures, on older patients as time goes on (Figures 3, 4). One possible explanation is the relatively higher frequency of nerve transfer procedures in general when compared to plexus dissections. Surgeons may be more familiar with these procedures and thus more comfortable offering them to older NBPP patients. Another reason for associations between increasing age and type of surgery could be that surgeons wait longer with patients where nerve transfers would be performed to better assess the quality of donor nerves, making the decision about surgery easier. Better obstetric care leads to decreasing numbers of patients presenting with complete palsies, potentially leaving the surgeons used to perform technically easier NtwoRE procedures unprepared to tackle such cases, where difficult exploration of the brachial plexus is necessary.

The emergence and success of acellular processed nerve allografts in treating NBPP (76) may exert an opposing influence by obviating the need for donor nerve harvest or changing favored surgical approaches. These products' impacts should be followed closely in the future.

## Limitations

This review included published studies only. It does not explore the full spectrum of surgeons' preferences and should be followed by a worldwide survey. Additionally, reliance on published information limited our classification of surgical approach to descriptions provided in the methods section of each paper. There were also numerous articles for which IPD was unavailable, even after attempts to contact the authors. The exclusion of these articles may have biased our results. Interpreting our results is complicated by the variability of NBPP severity that we included. We included any operative brachial plexus lesion; thus our results do not capture lesion-specific trends. Many surgeons tailor their surgical approach to the specific location of a lesion, and if there are geographic or time trends in these approaches, we are unable to detect them. The usage of publication date as the year of record also complicates our analysis. Many investigations into NBPP occur over the course of years and are published years after data collection is finished. Thus, the use of publication dates may present trends in a delayed fashion. Interpretation of data from Africa and South America is limited by the small number of included studies and patients from these areas. Finally, we analyzed trends in procedures targeted at NBPP, but not the specific deficit resulting from NBPP, largely due to the limited number of eligible papers for any given deficit. Thus, trends in procedures targeted at restoring deficits in, for example, elbow flexion vs. those targeted at restoring hand function are grouped together. More granular, deficit-based trends are not apparent from our data.

## Conclusions

Age at the time of surgery for NBPP is increasing over time. Technically easier surgical approaches to NBPP surgery avoiding exploration of roots of brachial plexus are becoming more popular. Regional differences exist in the age at surgery for NBPP. North and South America tend toward later intervention while Asia and Europe favor earlier intervention. NTwoRE was most common in North America. EBP is more common in younger patients while NTwoRE is more common in older ones.

## Data Availability

The raw data supporting the conclusions of this article will be made available by the authors, without undue reservation.
